# Characterisation of Electrical and Stiffness Properties of Conductive Textile Coatings with Metal Flake-Shaped Fillers

**DOI:** 10.3390/ma12213537

**Published:** 2019-10-29

**Authors:** Veronica Malm, Fernando Seoane, Vincent Nierstrasz

**Affiliations:** 1 Textile Materials Technology, Department of Textile Technology, Faculty of Textiles, Engineering and Business, University of Borås, 50190 Borås, Sweden; 2 Institute for Clinical Science, Intervention and Technology, Karolinska Institutet, 171 77 Solna Stockholm, Sweden; 3 Department of Medical Care Technology, Karolinska University hospital, 141 57 Huddinge, Sweden

**Keywords:** conductivity, metal flake, coating, e-textile, encapsulation, durability, stiffness

## Abstract

Two conductive formulations containing different types of micron-sized metal flakes (silver-coated copper (Cu) and pure silver (Ag)) were characterised and used to form highly electrically conductive coatings (conductors) on plain and base-coated woven fabrics, the latter in an encapsulated construction. With e-textiles as the intended application, the fabric stiffness, in terms of flexural stiffness and sheet resistance (R_sh_), after durability testing (laundering and abrasion) was investigated and related to user friendliness and long-term performance. Bare and encapsulated conductors with increasing amounts of deposited solids were fabricated by adjusting the knife coating parameters, such as the coating gap height (5, 20, 50, and 200 μm), which reduced the R_sh_, as determined by four-point probe (4PP) measurements; however, this improvement was at the expense of increased flexural stiffness of the coated fabrics. The addition of a melamine derivative (MF) as a cross-linker to the Cu formulation and the encapsulation of both conductor types gave the best trade-off between durability and R_sh_, as confirmed by 4PP measurements. However, the infrared camera images revealed the formation of hotspots within the bare conductor matrix, although low resistances (determined by 4PP) and no microstructural defects (determined by SEM) were detected. These results stress the importance of thorough investigation to assure the design of reliable conductors applied on textiles requiring this type of maintenance.

## 1. Introduction

Metals have attracted increasing interest as conductive fillers in textile coatings and printing formulations for implementing textile-electronic integration in e-textiles and, in particular, in wearable measurement applications [1,2,3]. The interest is likely to depend on the superior electrical conductivity characteristics of metals [4], which is also why metal fillers are the focus in this experimental research. Note that carbon black, intrinsically conductive polymers [5] and graphene [6] are the promising alternatives. High and reliable electrical conductivity is often required for power distribution or for interconnecting leads for sensors or transducers, e.g., textile electrodes for current stimulation, biopotential sensing [7,8], etc. Electrical conductivity depends on the ability of electrons and ions as charge carriers to move through the material—the deposited film matrix in this case; therefore, solid-to-solid contacts between metal fillers forming a three-dimensional (3D) conductive network embedded in the insulating polymeric matrix is of paramount importance to obtain conductivity in the deposited film. 

Compared to smaller metal particles, micron-sized flakes typically have a high aspect ratio, which means that less filler is required to generate enough contacts between flakes to achieve a sufficient conductive pathway [3]. However, the size of the filler also comes with certain drawbacks, such as increased stiffening effects when deposited on flexible fabrics. Less flexible fabrics and brittle conductive networks are prone to cracking upon bending [7,9,10], which adversely affect the comfort of the wearer and the long-term usage (durability) due to the loss of conductivity. Since these types of conductive fillers possess superior conductive properties, micron-sized flakes as metal fillers still remain a current research topic [2], specifically in terms of overcoming the associated challenges [11,12,13]. 

The encapsulation of conductive films (conductors) on textile substrates is a promising approach to protect from material fatigue caused by mechanical stress [11,12,14] and chemical attacks, and for some applications, this is the only alternative to prevent electrical short circuits between conductors and surface oxide film formation. In terms of environmental impact, encapsulation should also prevent the release of metal particulates from functionalised metal fabrics into sweat [15] or washing effluents [16,17], which may have a potential risk to humans [18] and other biological organisms [19]. Komolafe et al. [14] supposed that the encapsulation technique may be helpful for protecting the conductor from abrasion, although it does not structurally optimise the stress response of the conductor. Stress developed during processing may lead to a change in the layer curvature and misalignment between layers, which can cause severe reliability problems in microelectronic devices [20]. Therefore, the performance of encapsulated conductors applied on flexible substrates is important, which should be addressed when working with conductive formulations and their application on textiles, as done in this study. 

Furthermore, for certain applications, exposed conductors can be desirable, e.g., when skin-electrode interaction is desired, and the addition of a cross-linker to a conductive formulation to strengthen the film matrix is a possible approach to improving the durability. Melamine cross-linkers as additives for waterborne coating formulations have been extensively studied for their ability to improve the mechanical and chemical resistance of industrial coatings [21]. However, to the best of the authors’ knowledge, the addition of a melamine derivate to a waterborne polyurethane formulation containing metal flakes has not been addressed in any recent paper. 

Though encapsulation or cross-linking may be beneficial for the durability in terms of the conductive performance, there is also the risk of counteracting effects, such as increased stiffness, weight, and brittleness, which influence the comfort and user friendliness. These effects can be caused by a substantial increase in the amount of applied material or excessive cross-links tying the polymer chains together, resulting in increased stiffness. Therefore, a trade-off between fabric stiffness and flexibility must be addressed to improve the durability of the conductive network while retaining the flexibility of the fabric.

In this experimental research, conductive formulations containing metallic flake-shaped fillers coated on fabrics in a bare and an encapsulated construction are studied. First, the different coating formulation compositions, coating conditions and parameters to adjust the shear rate and viscosities are confirmed. These factors are then linked to the physical properties of the conductors and further related to the electrical and mechanical properties. Second, the morphological and electrical properties correlating to the factors and their relation to the durability during washing and abrasion are analysed. Finally, infrared images of the electrical conductors are obtained, and the optimisation of reliable conductors is discussed in this work. This work provides a guideline for how to characterise bare and encapsulated coated, printed or alternatively manufactured conductive fabrics using conventional textile test methods and a straightforward electrical resistance measurement method. 

## 2. Materials and Methods 

### 2.1. Materials

#### 2.1.1. Substrate

A plain-woven fabric (Almedahl-Kinna AB, Kinna, Sweden) made from poly(ethylene terephthalate) (PET) fibres, scoured and heat-set by the supplier, was used as the substrate. The fabric was composed of a continuous multifilament yarn, with a thread count of 30.5;24 and a mass per unit area of 110 g/m^2^.

#### 2.1.2. Coating Formulation

This study includes three formulations containing metal flakes as the conductive filler component. One is a water-based polyurethane (PU)-compound containing an emulsifier agent, thickening agents and silver-coated copper flakes with an average diameter of <12 μm (denoted Cu). The Cu formulation had a solids content of approximately 66 wt% and a density of 1 g/cm^3^ according to the supplier (Product U1005, Bozzetto Group, Italy). The Cu formulation was investigated with the addition of a cross-linker (3 wt%) based on a melamine derivative (MF) with a minimum content of free formaldehyde (Reacel MFC, Bozzetto Gmbh Krefeld, Germany) (denoted Cu_MF_). Third, a stretchable formulation containing pure silver flakes <5 μm (PE872, DuPont Electronic Materials Ltd., Bristol, UK) (denoted Ag) was included in the study. The Ag formulation had a solids content of 61 wt%, as determined by the author by differentially weighing the formulation before and after drying according to the conditions in Table 1. 

For the interfacial layer between the fabric substrate and the conductor, an aqueous aliphatic polyether polyurethane dispersion (RUCO-COAT PU 1110, Rudolf GmbH, Geretsried, Germany) thickened with a rheology modifier (Borchi Gel L75N, OMG Borchers GmbH, Langenfeld, Germany) was used as a basecoat. To encapsulate the conductor, an elastic thermoplastic polyurethane film (TPU) (LB700-100, Loxy AS, Halden, Norway) was used.

#### 2.1.3. Sample Manufacturing

**Figure 1:**
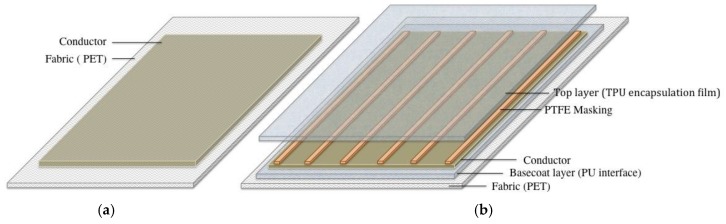
Illustration of the coated samples with different constructions: (**a**) conductor on plain fabric and (**b**) layered construction of an encapsulated conductor with PTFE masking prepared for washing treatments.

Figure 1a shows and illustration of coated samples with the conductor applied directly on top of the fabric (series: Cu_05, Cu_20, Cu_50, and Cu_200; Cu_MF__05, Cu_MF__20, Cu_MF__50, and Cu_MF__200; and Ag_05, Ag_20, Ag_50, and Ag_200), sample notations are further explained in Table 1. Fabric substrates of 20 × 30 cm^2^ were attached to an even surface, and the formulation was manually applied using a film applicator (ZUA 2000, Zehntner GmbH Testing Instruments, Sissach, Switzerland) using a procedure similar to the knife-over-blanket technique. Samples with four different gap heights (*h*) of 5, 20, 50, and 200 μm were prepared with a coating speed (v) of 0.6 m/min. The corresponding shear rates, drying and annealing conditions in a low convection heat oven (D-36-03-EcoCell, Rycobel NV, Deerlijk, Belgium) are shown in Table 1. The shear rate $\dot{\gamma }$ was calculated according to:

(1) $$\dot{\gamma }=\frac{v}{h}$$

Encapsulated conductive samples (Series: B_Cu_MF_05_T, B_Cu_MF_20_T, B_Cu_MF_50_T and B_Cu_MF_200_T; and B_Ag05_T, B_Ag20_T, B_Ag50_T, and B_Ag200_T) were prepared in the four-layer construction shown in Figure 1b. A basecoat was first applied to the fabric with the knife-over-blanket technique, as described above, with an applicator gap height of 100 μm. The basecoat was then dried at 90 °C for 20 min without an annealing step to increase the adhesion to the coated conductive layer applied in the next step. The conductive layer was dried and annealed before the top layer was applied, see Table 1. To expose the conductors for electrical resistance characterization, a polytetrafluoroethylene (PTFE) mask with rectangular stripes was applied on the conductive layer (see Figure 1b) before the top-layer TPU film was laminated using a hot-press plate set at a temperature of 130 °C for 1 min and 40 s. 

### 2.2. Characterisation

#### 2.2.1. Viscosity

The viscosities of the conductive and basecoat formulas were measured using a Paar Physica MCR 500 rheometer (Anton Paar, Ostfildern, Germany) at 23 °C with a cone and plate setup at a distance of 50 μm. Three measurements of each formulation were taken at shear rates between 0.01 and 2000 s^−1^, under simulated process conditions during the coating procedure. The shear rates are listed in Table 1.

#### 2.2.2. Surface Topography and Morphology 

The surface topography (or surface roughness) of coated samples was evaluated by a non-contact (optical) high-resolution surface profiler (Wyko NT 1100, Veeco Instruments Inc. Tucson, AZ, USA). The arithmetical mean deviation of the profile, (R_a_), with the dimensional unit of micrometre, was obtained from 2D roughness measurements in the vertical z direction. The surface morphology (visual appearance) was studied by means of scanning electron microscopy (SEM-JEOL JSM 6301F, JEOL Ltd., Tokyo, Japan) of the sputtered samples.

#### 2.2.3. Flexural Stiffness

The conventional flexural stiffness of the coated samples was characterised by a fixed-angle flexometre according to the SS-ISO 4604:2011. The test was performed by sliding a conditioned (12 h, 22 °C, 65% RH) sample (25 × 200 mm^2^) over the edge of a horizontal surface until the tip bends under its own weight and makes a 41.5° angle to the horizontal by touching the inclined surface. The length of the overhang was measured, and the tests were made in both weft and warp direction and were repeated on both ends in face up and down orientations with a total of four readings per sample. The conventional flexural stiffness (G) in millinewton metres was calculated according to the standard, and the average flexural stiffness from four replicates was used. 

#### 2.2.4. Electrical Resistance

**Figure 2:**
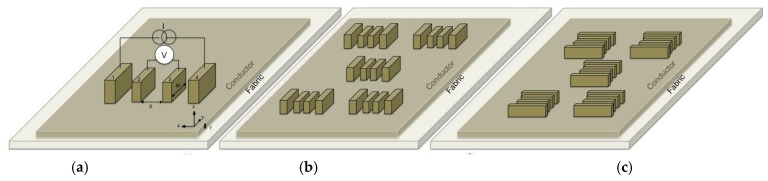
(**a**) Four-point probe setup and the placement of the probe at five different positions in the (**b**) weft and (**c**) warp directions in the 4PP arrangement.

The electroconductive properties were evaluated based on an electrical resistance technique, which involved the inline 4PP arrangement using a probe with four rectangular electrodes arranged as shown in Figure 2a and further described elsewhere [22]. The electrode surface was coated with a textured silver conductive epoxy layer, and a weight of 2 kg was placed on top of the probe before each measurement to obtain better contact with the coated textile surface. For plain conductors, the probe was consequently placed contacting the surface of the conductor at five positions on each rectangular sample in both the weft (x) and warp (y) direction, as shown in Figure 2b,c. To enable measurement on the encapsulated samples and because no significant resistance anisotropy was found when using the 4PP arrangement, measurements on the encapsulated samples were taken only in the weft direction. 

Electrodes were connected to a multimetre (Agilent 34401A) set in four-wire resistance mode with a low test current flow of 1 mA and an accuracy of ±4 mOhm. The values were recorded after 1 min to circumvent initial fluctuations. The outer electrodes were current (I) carrying, and the inner electrodes (forming a 20 × 20 mm^2^ square) were voltage (V) sensing. The values are presented as sheet resistance (R_sh_) according to:

(2) $$R=R_{sh\;}\frac{L}{W}=\frac{\rho }{t}\frac{L}{W}$$

**Figure A1:**
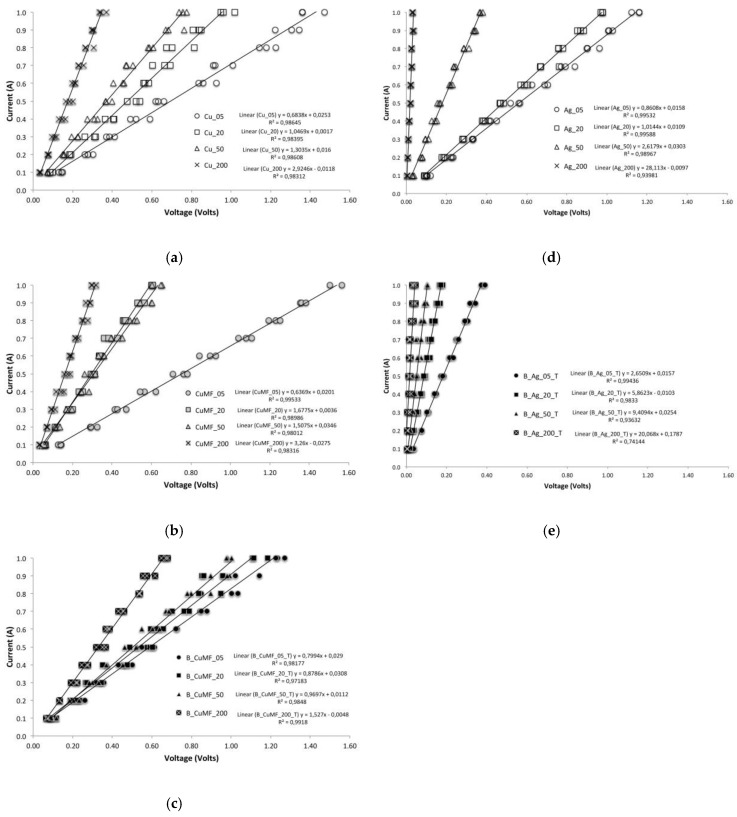
Current versus voltage curves (**a**) for sample series: Cu_05, Cu_20, Cu_50, and Cu_200; (**b**) for samples series: Cu_MF__05, Cu_MF__20, Cu_MF__50, and Cu_MF__200; (**c**) for samples series: B_Cu_MF_05_T, B_Cu_MF_20_T, B_Cu_MF_50_T and B_Cu_MF_200_T; (**d**) for samples series: Ag_05, Ag_20, Ag_50, and Ag_200; and (**e**) for sample series: B_Ag05_T, B_Ag20_T, B_Ag50_T, and B_Ag200_T.

To validate the ohmic linearity for all coated samples current–voltage (IV) curves were obtained using the described arrangement with an additional power supply and by varying the current from 100 up to 1000 milliamperes, see Figure A1 in Appendix A. 

### 2.3. Durability Test

#### 2.3.1. Washing

Durability to laundering was studied according to the standard EN ISO 6330:2012 method 4G with a ballast load of 1 kg (Type III) using a 40 °C hand washing procedure with detergent in a laboratory washing machine (Electrolux Wascator FOM71 MP, Stockholm, Sweden). To avoid samples from getting stuck in the drum, the samples were attached to ballasts using tag fasteners with the coated surface facing outward. Five replicates were characterised for their electrical resistance before and after washing using the four-point probe method. The influence of the drying procedure between washing cycles was studied, and line drying at room temperature (24 °C) for 24 h and line drying in a heat cabinet at 50 °C for one hour were compared.

#### 2.3.2. Abrasion

The impact of abrasion on the coated samples with one conductive layer was tested with a Martindale 2000, using the standard SS-EN ISO 5470-2 method 2 and dry tests. The influence of abrasion on the electrical resistance was evaluated by performing 4PP measurements in the warp and weft direction on three replicates. For both plain and encapsulated samples 4PP measurements were made before and in between each inspection stage, that is, after 1600, 3200, 6400, 12,800, 25,600, and 51,200 revolutions corresponding to 100, 200, 400, 800, 1600, and 3200 abrasion cycles. For the encapsulated samples, electrical measurements were only made in the weft direction and on three different replicates from the same sample series in between each inspection stage. 

## 3. Results and Discussion

### 3.1. Influence of the Formulation Type and Coating Parameters on the Physical, Electrical and Mechanical Properties

#### 3.1.1. Viscosity Behaviour of the Formulations

**Figure 3:**
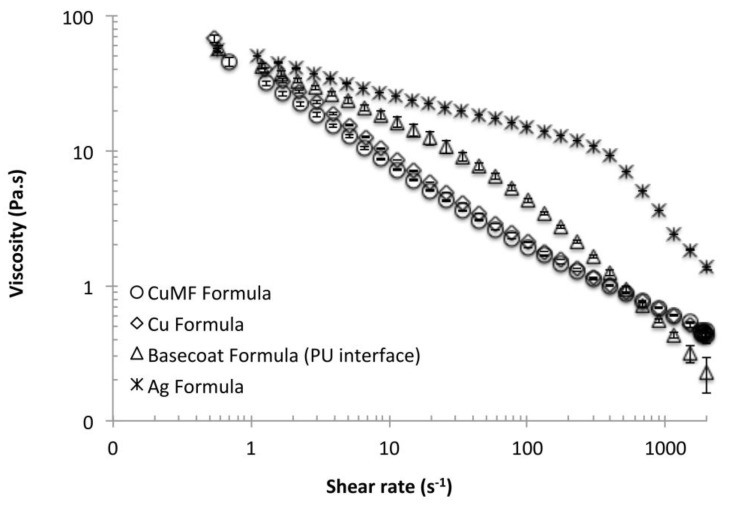
Viscosity versus shear rate for the three conductive formulations and the formula used at the interface.

Figure 3 shows the viscosity versus shear rate for the conductive formulations listed in Table 1, including the formulation used for the interface layer between the textile substrate and the conductor layer. For all formulations, the viscosity decreased with increasing shear rate related to the coating conditions in Table 1, demonstrating pseudoplastic behaviour. At shear rates below 10 s^−1^, the addition of the MF had a slight dilution effect on the Cu_MF_ formulation, producing a lower viscosity than that of the plain Cu formulation. However, with increasing shear rates (50, 200, 500 and 2000 s^−1^) by tuning the application conditions, the differences were suppressed, resulting in overlapped curves. The differences at the lower shear rates are more relevant for the film-forming process after application, where a lower viscosity offers increased levelling conditions, whereas viscosity differences at higher shear rates can theoretically influence the amount of solids deposited. 

At lower shear rates, the viscosity of the Ag formulation was higher than that of the Cu and Cu_MF_ formulations, and with increased shear rates up to 300 s^−1^, the shear thinning behaviour was less steep, showing a more Newtonian profile, i.e., the viscosity was less influenced by the shear rate. This indicates that the binder polymers within the Ag formulation are of high molecular weight and long enough that polymer entanglement restricts their alignment in the flow direction with increased shear rates up to 300 s^−1^. However, at increase shear rates, a more pseudoplastic behaviour was pronounced.

#### 3.1.2. Influence of Solids Deposit on Surface Topography and Morphology

**Figure 4:**
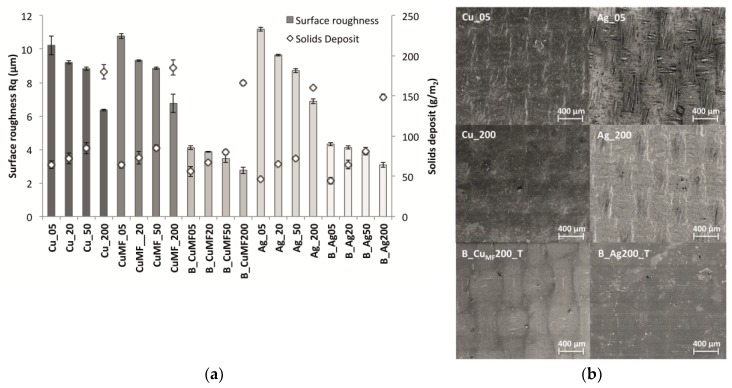
(**a**) Surface roughness of the conductor layers and solids deposition depending on the formulation and gap height. Individual standard deviations were used to calculate the intervals, and the plot displays a 95% confidence interval (CI) for the mean. (**b**) Scanning electron microscopy (SEM) images of samples coated with the Cu and Ag formulations showing the mass per unit area in brackets Cu_05 (64 g/m^2^), Cu_200 (180 g/m^2^), B_Cu_MF_200_T (166 g/m^2^), Ag_05 (47 g/m^2^), Ag_200 (160 g/m^2^) and B_Ag200_T (149 g/m^2^).

In Figure 4a the surface roughness (R_a_) values were observed to decrease with an increased amount of solids (because of the gap height). This was because the increased amount of deposited solids levelled out the surface topography of the fabric. The phenomenon is also what caused the significant difference in R_a_ between the plain-coated and base-coated sample series, the value of the latter decreasing to half of the value of the former. This is supported by the SEM images in Figure 4b showing the surface morphology differences of Cu- and Ag-coated samples produced with the lowest (5 μm) and the highest (200 μm) coating gaps, including the encapsulated samples with the highest coating gap. 

The basecoat has a levelling effect on the fabric texture before the conductor is applied, which also hinders the formulation from penetrating the cavities between the yarns. The total decreases in R_a_ with increased solids deposition is therefore not as pronounced (<1.3 μm) for the base-coated sample series compared to the plain-coated samples, which decreased by over 4 μm. Thus, a more homogenous morphology can be expected for base-coated conductor layers with less variation in the surface topography. However, an undulated microstructure conductive layer is still visible on the surface of the encapsulation film, even on the base-coated samples, as seen in Figure 4b. 

It is important to note that surface roughness is a material property that may influence electrical resistance measurements since the contact between the conductor layer and the electrodes of the measurement probe likely contribute to the electrical contact resistance. 

No significant difference in R_a_ was seen between the Cu and Cu_MF_ samples, since there was also no difference in the amount of deposited solids, see Figure 4a. This correlates well with the equivalent pseudoplastic behaviour of the Cu and Cu_MF_ formulations, as seen in Figure 3. Accordingly, equivalent penetration of the formulation into the woven structure can also be assumed. When comparing the plain-coated and encapsulated Cu_MF_ sample series with the same gap heights, a trend towards less solids deposition for base-coated fabrics was observed. This is the result of the preventative effect of the formulation flowing into the fabric, as discussed above. 

A lower amount of solids was deposited on the Ag-coated samples than the samples coated with the Cu and Cu_MF_ formulations. The plain coated and base-coated Ag samples showed the same trends. However, the Ag formulation showed a higher viscosity at the relevant shear rates depending on the processing conditions (see Table 1), which generally leads to a larger amount of applied material. The reason for this is most likely the lower solids content in the Ag formulation (61 wt%) than in the Cu formulation (66 wt%). Furthermore, the concentration of metal flakes in each formula should be taken into account, although this information is unknown to the authors. Though the filaments in the yarn appear on the surface of Cu_05 show a rather coarse texture, a coherent film without cracks or blisters was achieved. However, for Ag_05, the deposited amount of 47 g/m^2^ was not enough to achieve a coherent film, as the filament yarn was only partly covered with the material. On the other hand, with an increased amount of deposited material, as for Ag_200, the film was more properly covered.

#### 3.1.3. Influence of Solids Deposit on Electrical Resistance

**Figure 5:**
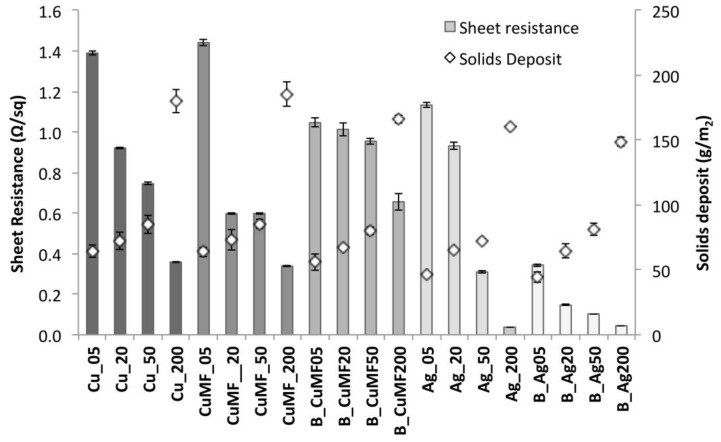
Sheet resistance and solids deposition for all samples using the inline four-point probe configuration. In this diagram, the thickness of the encapsulated conductor is not taken into account; thus, the R_sh_ instead of the bulk resistivity is displayed.

Figure 5 shows the R_sh_ as a function of solids deposit. For all samples, the R_sh_ values are below 1.5 Ω/sq, indicating that all of the conductor layers possess high electroconductive properties. A significant decrease in R_sh_ with increased solids deposit (because of increased coating gap) from 44 to 185 g/m^2^ was observed and attributed to the increased number of flakes within the polymer matrix, creating more contact points between flakes and more possible routes for electron conduction through the 3D network. A similar decrease in R_sh_ is seen for the Cu and Cu_MF_ curves on plain fabrics, which can be correlated to the similarities in increased solids deposition. The decreasing trend is, however, not as pronounced for the B_Cu_MF__T curve, and no significant difference in R_sh_ between the plain-coated and encapsulated conductor was noticed, even though there was a clear difference in solids deposition between the samples, especially those with the largest amount (Cu: 180, Cu_MF_: 185 and B_CuMF_T: 166 g/m^2^). The observed phenomena may be related to the more uniform microstructure of the conductor applied on the base-coated samples, which facilitates electron transport pathways, resulting in a low R_sh_ with a lower amount of deposition. On the other hand, conductors on plain fabric conform to the substrate texture, resulting in an undulated film with increased electron path lengths, and the equal R_sh_ values were possibly compensated by the increased deposition. 

Compared to the Cu sample, the Ag sample showed a more pronounced decrease in R_sh_ with increased deposition, with a total decrease of more than one order of magnitude from 1.1 to 0.03 Ω/sq. The explanation for this is that silver possesses a lower intrinsic resistivity than copper, 16 and 17 nΩ m respectively [4]. It was interesting to see that Ag_05 had a mean R_sh_ value of 1.2 Ω/sq, although the SEM images in Figure 4b revealed a non-coherent topography. This result indicates that the Ag formulation penetrates the fabric and forms a conductive network between the interstices of the yarn filaments. 

#### 3.1.4. Influence of Solids Deposit and Construction on Fabric Flexibility

**Figure 6:**
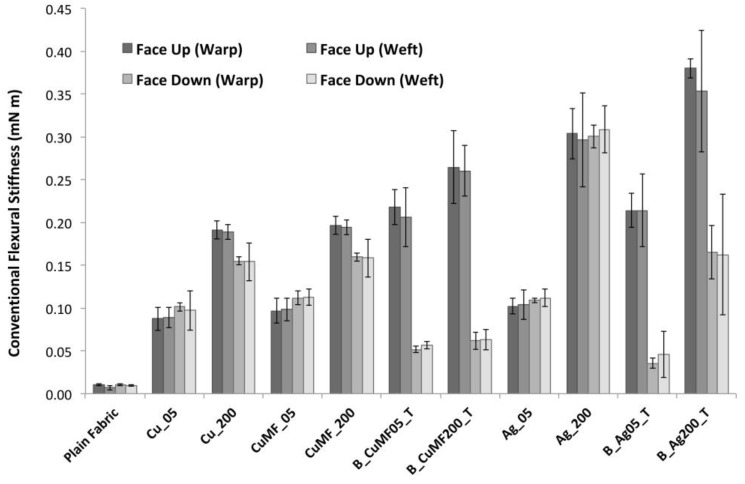
Conventional flexural stiffness of plain and coated fabrics with the lowest and highest amount of deposited solids (received from gap heights of 5 and 200 m) in the warp and weft direction and whether the sample is in the face up or down orientation. Individual standard deviations were used to calculate the intervals, and the plot displays a 95% CI for the mean.

The fabric stiffness in terms of conventional flexural stiffness of the plain and coated fabrics with the lowest and highest amount of solids deposition (generated from a gap height of 5 and 200 μm) is presented in Figure 6, showing average values in the warp and weft direction, as well as in the face up and down orientation. For the plain fabric, no significant difference in the flexural stiffness in the warp and weft direction or the face up or down orientation of the samples was observed. This means that fabric anisotropy because of differences in thread count did not influence the fabric stiffness. Furthermore, it shows that the addition of the MF did not impact the fabric stiffness, as the confidence intervals overlapped for the Cu and Cu_MF_ samples within the same gap span. For all samples, increased deposition lead to a substantial increase in flexural stiffness, as shown by the flexural stiffness values starting from ~0.01 mN m for plain fabric and increasing by a factor of 10 for the samples with the least deposition and almost doubling for the samples with the most deposition. The layered B_Ag200_T samples had the highest flexural stiffness values of ~0.38 mN m. Most certainly, this stiffening behaviour would decrease the drapeability and comfort, even when these types of formulations are printed instead of coated as in this study, provided that the same amount of deposition material is used. 

For this study, it was interesting to differentiate between the faces of the fabric, especially since fabric flexibility leads to perturbations to the electrical potential within a conductor. Flexibility in the context of conductors is a counterproductive property, where higher flexural stiffness will reduce the dimensional changes upon bending, having less impact on the conductive network. This is to reduce perturbations within the conductor, whereas lower flexural stiffness is preferred to enhance the drape and comfort for the wearer. Therefore, for closer examination, the flexural stiffness of the fabric samples in both the face up and face down orientations are plotted in Figure 6. A significant difference depending on fabric face orientation was observed for samples with a larger amount of coating (except for Ag_200), and the differences further increased for samples with a layered construction. The higher values of the face up orientation reflect the enhanced resistance to bending and are relative to the larger amount of applied material, forming a stiffer fabric surface. Lower values are seen for fabrics in the face down orientation, and with an increased amount of applied material, it is evident that these samples bend more easily under their own weight, resulting in a lower stiffness. This trend applies when the majority of the coating stays on the fabric surface and does not severely penetrate the fabric construction, as in the case of Ag_200, of which the flexural stiffness is equal regardless of whether the coated fabric is face up or down. The reason for this is likely that the polymer diffused inside the fabric construction, resulting in the mechanical interlocking of the woven yarn construction because of the penetration of the applied viscous formulation. For the Ag_200 sample and in the face down orientation, the resistance to bending was even slightly larger than that of the layered B_Ag200_T sample, despite the fact that the latter had an increased amount of applied material. 

### 3.2. Influence of Durability Testing on the Morphology and Electrical Properties

#### 3.2.1. Influence of Washing on the Morphology

**Figure 7:**
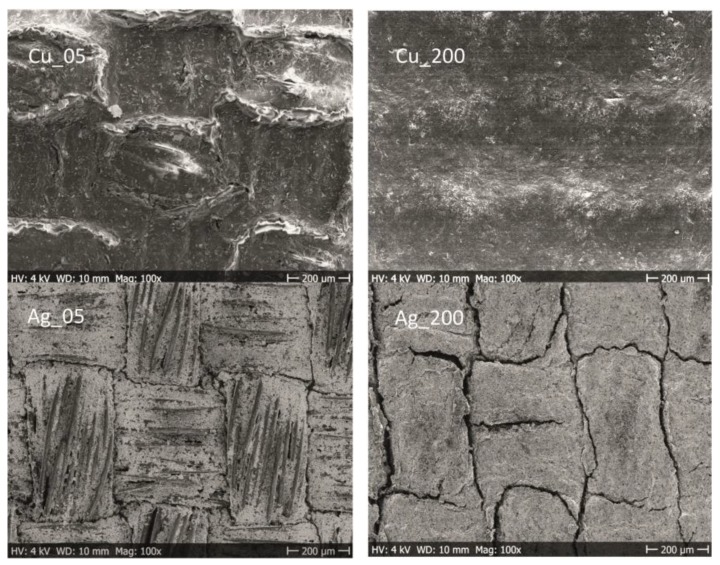
Top-view SEM images showing the microstructures of the coated samples after five washing cycles.

In Figure 7, the SEM images show the surface appearance of plain Cu- and Ag-coated samples after five washing cycles. These samples were produced with the lowest (5 μm) and the highest (200 μm) coating gaps and showed major differences in appearance. The encapsulated samples did not visually show any significant differences before or after the washing procedure and are therefore not included in this section. In general, the washing procedure affected the surface appearance of the plain-coated fabrics to a large extent. The Cu_05 samples with the lowest deposition displayed a film topography with a larger degree of roughness compared to the untreated sample (for comparison, see Figure 4). The surface showed an embossed character where the boundaries between the filament yarns are constituted. Apparently, this is a combined effect from the harsh mechanical treatment in the drum and the chemical and wetting processes during the washing cycles. A similar topography was seen for samples with the lowest deposition and containing the cross-linker; thus, the addition of a MF did not visually influence the conductor resistance to washing. However, with increased deposition, as seen for washed Cu_200, the topography was unaffected and was similar to the untreated sample. One can argue that thicker conductive coatings with an increased amount of deposited solids and a smoother topography generally withstands the mechanical abrasions in the drum to a larger extent. The SEM images of the Ag-coated topography show clear visible cracks for both Ag_05 and Ag_200. Increased amounts of Ag formulation did not improve the resistance to washing. 

#### 3.2.2. Influence of Washing on the Electrical Resistance

**Figure 8:**
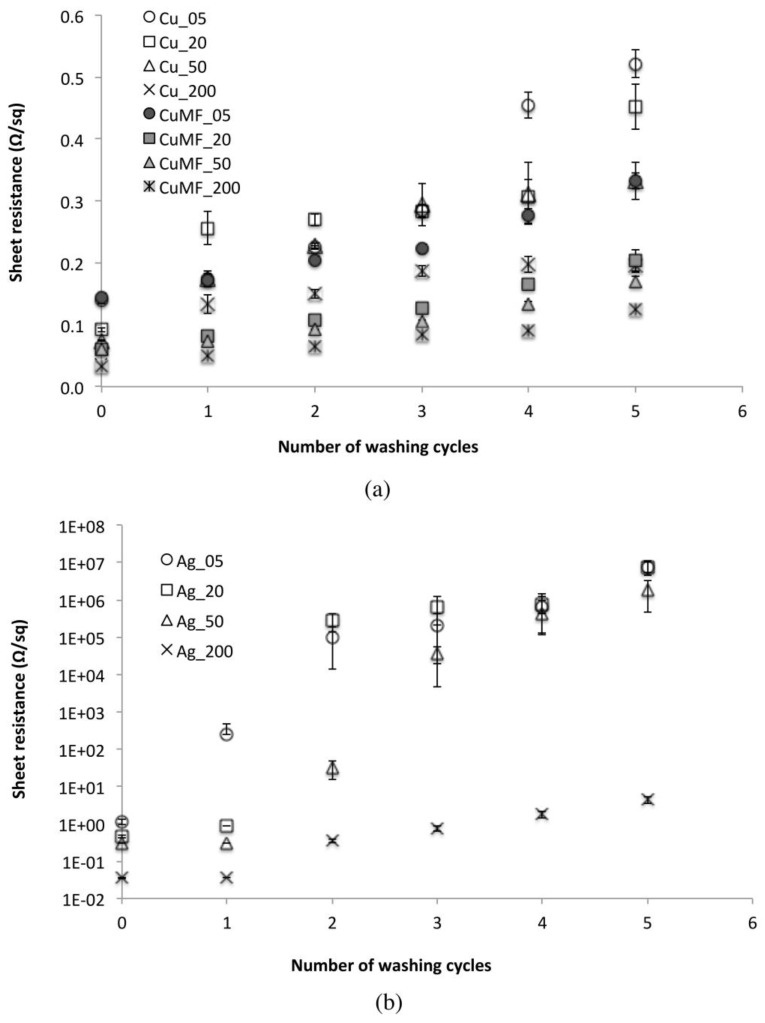
Sheet resistance of (**a**) the plain Cu and Cu_MF_ formulation and (**b**) the Ag-coated samples before and after five washing cycles. Error bars represent 95% CI for the mean, and individual standard deviations were used to calculate the intervals.

The influence of washing on the R_sh_ for the plain samples is seen in Figure 8. The R_sh_ value for both the Cu and Cu_MF_ conductor layers (see Figure 8a) increased with the number of washing cycles, and after five cycles, these layers resulted in values below 6 and 3.3 Ω/sq, respectively. However, the Cu_MF_ samples were shown to be less affected by washing, and because of the similarities in terms of the amount of deposited solids and the penetration of the formulation in Cu- and Cu_MF_ samples (see Table 1 and the morphology section), this deviation could be related to the different network structures. The Cu_MF_ matrix likely has a larger degree of polymer chain interdiffusion or even chemically cross-linked polymers than the Cu matrix without the addition of a MF. Nevertheless, the infrared camera images in Figure 8a reveal the formation of hotspots within the conductor, even though the resistance of the conductor to washing in terms of the R_sh_ was reasonably good and the microstructure was seemingly intact (based on the SEM images in Figure 7). Most likely, the mechanical stress exerted on the coated textile during washing lead to reduced contact between flakes, resulting in capacitive junctions within the matrix, where a constriction of the charges causes substantial heating and the possible risk of conductor malfunction. 

For the plain Ag layers applied using coating gaps from 5 to 50 μm, there was a sharp increase in the R_sh_ with increased washing cycles from one to five cycles, see Figure 8b. This fragile behaviour is clearly the result of cracking of the layers after the washing treatment, as displayed in the SEM images in Figure 7. However, the Ag_200 samples applied with the largest amount, maintained values below 5 Ω/sq after five washing cycles, despite the appearance of visible cracks in the microstructures of these samples. This means that the Ag flake network was maintained at several connection points within the polymer matrix closer to the fabric. Still, the application of increased amounts of Ag formulation obviously did not improve the resistance of the microstructure to washing, which supports the idea that this type of Ag formulation needs to be protected to obtain a stable and coherent conductor, at least when using the coating procedure followed herein. 

#### 3.2.3. Encapsulation and Drying in between Washing Cycles.

**Figure 9:**
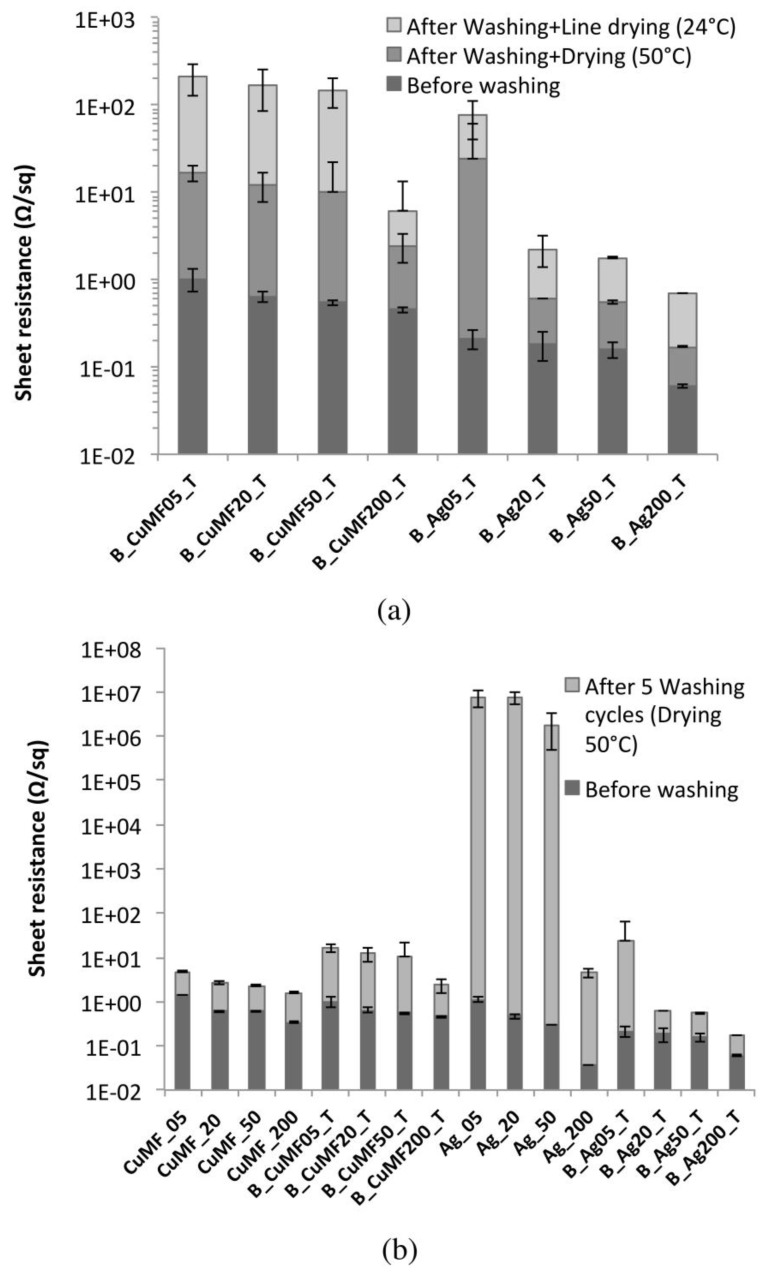
(**a**) Sheet resistance before and after different drying procedures in between washing cycles for the encapsulated samples and (**b**) the sheet resistance before and after five washing cycles with a 50 °C drying step for samples with and without encapsulation.

Figure 9a shows the R_sh_ of the encapsulated samples before and after five washing cycles using two different drying procedures: line-drying at 24 °C and a RH of 54% and at 50 °C in a heat cabinet. For the B_Cu_MF__T sample series, a significant decrease in resistance by more than 90% was noticed when increasing the drying temperature from 24 to 50 °C. For the B_Ag_T sample series, the resistance decreased up to 80% regardless of the increasing deposition amount. Possibly, the encapsulation or the interface layer material absorbs water during washing, and the line-drying procedure at 24 °C over 24 h is insufficient. Retained water could lead to dimensional changes and induce stresses in the conductor layer, thereby increasing the resistance, whereas increased drying temperatures (50 °C) promote water evaporation, minimising residual stresses within the conductor and leading to decreased resistance values.

The protective performance of conductor encapsulation was evaluated, and the R_sh_ of samples with and without encapsulation before and after five washing cycles are compiled in Figure 9b. When comparing the Cu_MF_ and B_Cu_MF__T sample series, there was a significant increase in R_sh_ after washing regardless of the deposited amount of formulation. The samples with the lowest amount of conductor (CU_MF_ 05 and B_Cu_MF_05_T) showed the largest difference, with values increasing from 3 to 16 Ω/sq. The difference decreased with an increased amount of formulation, and for CU_MF_ 200 and B_Cu_MF_200_T, the difference was only approximately 1 Ω/sq. Despite that encapsulation of the conductors resulted in increased R_sh_ after washing, it is important to show that encapsulation provided protection against mechanical damage of the conductor, as shown in the IR camera images inset in Figure 9b. Compared to the washed Cu_MF__200 sample, which showed uneven heat distribution because of hotspots within the conductor, the B_ Cu_MF_200_T sample showed an even heat distribution with no signs of hotspots in the encapsulated conductor. When comparing plain and encapsulated Ag-coated samples, it is clear that encapsulation of the conductor is required for protection during washing, as the results show a decrease in R_sh_ of more than five orders of magnitude for the encapsulated washed samples. 

#### 3.2.4. Influence of Abrasion on the Electrical Resistance

**Figure 10:**
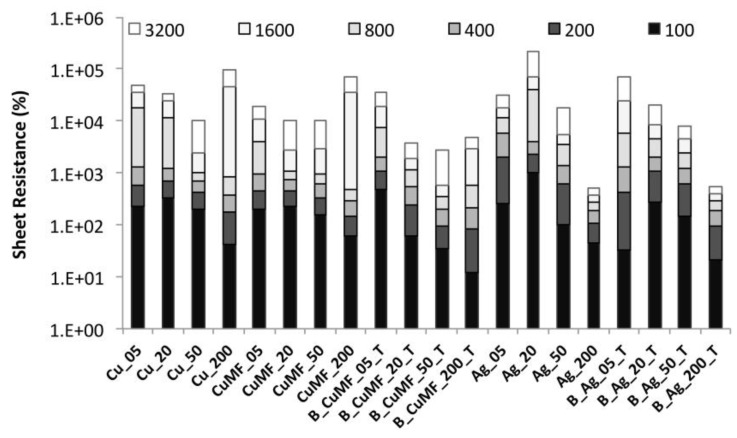
Sheet resistance before and after 100 to 3200 abrasion cycles for the plain and encapsulated conductors.

The effect of abrasion on the R_sh_ for samples with different formulations and layered constructions is shown in Figure 10. A trend in increasing R_sh_ with an increasing number of abrasion cycles is observed for all samples. For most samples, the total increase in R_sh_ after 3200 abrasion cycles was between two to three orders of magnitude, except for the silver-coated samples with a larger amount of coating (Ag_200 and B_Ag200_T), where the increase was below one order of magnitude, maintaining values below 0.4 Ω/sq. For the plain-coated Cu and Cu_MF_ samples, the R_sh_ values did not significantly differ with increased abrasion cycles depending on the added cross-linker. This means that neither the physically dried nor the possibly cross-linked conductor was sufficiently stable to withstand distortion from repetitive abrasion cycles in a meaningful way. Compared to the plain-coated Cu and Cu_MF_ sample series, the encapsulated samples showed a more gradual increase in R_sh_ with increased amount of conductor, depending on the gap height (5, 20, 50 and 200 μm) and the number of abrasion cycles. Furthermore, the total increase in R_sh_ was approximately 1.2 orders of magnitude, at least for the B_Cu_MF__T samples with an increased gap between 20–200 μm compared to the plain-coated samples within the same sample series for which the total increase in R_sh_ was closer to two orders of magnitude. Thus, the encapsulated conductor in the Cu_MF_ formulation with a solids deposition from 67 to 166 g/m^2^ (see Table 1) withstands abrasion cycles to a larger extent compared to the plain-coated conductors within similar ranges of solids deposition. 

A similar trend and a more gradual increase in R_sh_ with an increased amount of solids and number of abrasion cycles were observed for the encapsulated B_Ag_T samples. Compared to the plain-coated Ag formulation, the encapsulated Ag formulation also maintained R_sh_ values below 10 Ω/sq even after 800 abrasion cycles. Possibly, the encapsulation protects the conductor from abrasion up to a point with a solid deposition of approximately 80 g/cm^2^. However, with increased amounts over 150 g/cm^2^, there was no significant difference because of encapsulation, as the plain-coated and encapsulated samples with the largest amount of applied material showed similar behaviour. For both samples, the total increase in R_sh_ with increased abrasions was less than one order of magnitude, and the values were below 0.4 Ω/sq. 

For the plain-coated samples, no significant weight loss was detected after abrasion, although there was a visual fading and strengthening of the metallic gloss for the Cu- and Ag-coated samples, respectively, and the abrading cloth was slightly coloured. Therefore, the increased R_sh_ with increased abrasion cycles can only be partly explained by the loss of some of the polymer matrix and conductive material. Evidently, other reasons must also underlie the increasing R_sh_ trends, as displayed for the encapsulated samples as well. The results support that a balance must be found between the amount of applied formulation and the encapsulation to improve the resistance to abrasion. 

## 4. Conclusions

The obtained results confirm that both the addition of a cross-linking additive and encapsulation improved the durability of metallic conductive coatings and that increasing the amount of applied conductive material can reduce the R_sh_. An improvement in the durability and electrical conductivity was achieved by increasing the amount of deposited solids or by conductor encapsulation, but this was at the expense of increasing the flexural stiffness of the coated fabric. The flexural stiffness of the coated fabric increased by a factor of ten for samples with approximately 60 g/m^2^ of deposition, while it redoubled for samples produced with 170 g/m^2^ deposition. The flexural stiffness was also higher for samples in which the conductive polymer formulation diffused deeper into the fabric, interlocking the woven yarn construction, and for tested samples with the conductor fabric in the face up orientation. With a more rigid conductor, regardless of the coating method used, a less drapable coated fabric and perhaps also a less comfortable e-textile is obtained. More importantly, a more rigid conductor can be related to enhanced resistance to bending and prevention of dimensional changes, which can deteriorate the conductive network. The results showed that the largest amount of deposition and conductor encapsulation significantly improved the resistance to electrical fatigue after durability testing, which confirms that not only a trade-off between stiffness and flexibility of conductors must be considered but also that for certain conductive coatings, a high degree of stiffness is the only alternative to obtain durable electrical textile coatings. Note that the specific impact on comfort remains unknown and requires future studies targeting specific applications of e-textiles and wearable sensing. 

The results indicate that the addition of a cross-linking additive improved the electrical fatigue properties because of the formation of a stronger polymeric network. This was confirmed after excluding possible impacting factors such as viscosity, deposition amount and surface roughness. However, infrared camera images revealed hotspots within all the washed plain-coated conductor matrixes, even those with preserved low R_sh_ and without microstructural defects, as seen in the SEM images. The phenomenon was not seen for encapsulated conductors that had a more homogeneous morphology, because the conductive formulation was hindered from penetrating cavities between the yarn filaments. Given that trapped water within the bare conductor matrix can be excluded, the shearing forces during durability testing are suggested to have a larger impact on the bare conductive matrix than the encapsulated conductors, which were interlocked within the yarn construction, introducing small ruptures within the matrix and between the conductive flakes and leading to capacitor-type junctions, which at worst, can cause the conductor to malfunction. 

This paper confirms that conductive formulations applied to flexible substrates, such as textiles, must be extensively studied, specifically using durability tests, to ensure that the applied conductors are properly constructed with high and reliable conductive properties for long-term performance for e-textile applications. 

## Figures and Tables

**Table 1:** Coating conditions.

Sample	Shear Rate (Formulation)	Solids Deposit (Conductor)	Drying Temp:Time	Annealing Temp:Time
	(s^−1^)	(g/m^2^)	(°C:min)	(°C:min)
Cu_05	2000	64	80:5	150:2
Cu_20	500	72	80:10	150:3
Cu_50	200	85	80:15	150:4
Cu_200	50	180	80:20	150:5
CuCL_05	2000	64	80:5	150:2
CuCL_20	500	73	80:10	150:3
CuCL_50	200	85	80:15	150:4
CuCL_200	50	185	80:20	150:5
B_Cu_MF_05_T	2000	56	80:5	150:2
B_Cu_MF_20_T	500	67	80:10	150:3
B_Cu_MF_50_T	200	80	80:15	150:4
B_Cu_MF_200_T	50	166	80:20	150:5
Ag_05	2000	47	-	130:20
Ag_20	500	65	-	130:30
Ag_50	200	72	-	130:50
Ag_200	50	160	-	150:40
B_Ag05_T	2000	44	-	130:20
B_Ag20_T	500	64	-	130:30
B_Ag50_T	200	81	-	130:50
B_Ag200_T	50	149	-	150:40

## Appendix A

In Figure A1a–e current–voltage (IV) curves for all samples are shown. The data plotted are obtained from four points probe (4PP) measurements on finite samples and on three replicates. They all show a linear curve. The IV plotted data in all figures show positive slopes for conductive samples ohmic linearity.
